# Identification of novel candidate pathogenic genes in pituitary stalk interruption syndrome by whole‐exome sequencing

**DOI:** 10.1111/jcmm.15781

**Published:** 2020-08-31

**Authors:** Xuqian Fang, Yuwen Zhang, Jialin Cai, Tingwei Lu, Junjie Hu, Fei Yuan, Peizhan Chen

**Affiliations:** ^1^ Department of Pathology Ruijin Hospital Shanghai Jiao Tong University School of Medicine Shanghai China; ^2^ Department of Endocrinology Ruijin Hospital Shanghai Jiao Tong University School of Medicine Shanghai China; ^3^ Clinical Research Center Ruijin Hospital North Shanghai Jiao Tong University School of Medicine Shanghai China; ^4^ Department of Gastroenterology Shanghai General Hospital Shanghai Jiao Tong University School of Medicine Shanghai China

**Keywords:** hedgehog signalling pathway, pathogenic genetic variants, pituitary stalk interruption syndrome, whole‐exome sequencing

## Abstract

Pituitary stalk interruption syndrome (PSIS) is a type of congenital malformation of the anterior pituitary, which leads to isolated growth hormone deficiency or multiple hypothalamic‐pituitary deficiencies. Many genetic factors have been explored, but they only account for a minority of the genetic aetiology. To identify novel PSIS pathogenic genes, we conducted whole‐exome sequencing with 59 sporadic PSIS patients, followed by filtering gene panels involved in pituitary development, holoprosencephaly and midline abnormality. A total of 81 heterozygous variants, distributed among 59 genes, were identified in 50 patients, with 31 patients carrying polygenic variants. Fourteen of the 59 pathogenic genes clustered to the Hedgehog pathway. Of them, PTCH1 and PTCH2, inhibitors of Hedgehog signalling, showed the most frequent heterozygous mutations (22%, seven missense and one frameshift mutations were identified in 13 patients). Moreover, five novel heterozygous null variants in genes including *PTCH2* (p.S391fs, combined with p.L104P), *Hedgehog acyltransferase* (p.R280X, de novo), *MAPK3* (p.H50fs), *EGR4* (p.G22fs, combined with *LHX4* p.S263N) and *SPG11* (p.Q1624X), which lead to truncated proteins, were identified. In conclusion, genetic mutations in the Hedgehog signalling pathway might underlie the complex polygenic background of PSIS, and the findings of our study could extend the understanding of PSIS pathogenic genes.

## 1 INTRODUCTION

Pituitary stalk interruption syndrome (PSIS) is a congenital malformation of the anterior pituitary gland and usually presents in the imaging of a very thin pituitary gland or the complete absence of the anterior pituitary gland, an ectopic posterior pituitary gland, and with/without the truncated pituitary stalk.^1^ PSIS might not be diagnosed during the neonatal period or early infancy due to the lack of clear signs and symptoms. Most cases are diagnosed in childhood or adolescence due to growth retardation, the absence of secondary sex characteristics and infertility. PSIS is diagnosis mainly based on hormone level examinations and magnetic resonance imaging (MRI); however, the underlying mechanisms involved in PSIS ontogenesis have remained unclear. Perinatal injury including breech delivery, caesarean section and neonatal asphyxia is usually noticed in PSIS patients, which have been suggested as important aetiological factors of PSIS.^2^ Further, some studies suggested that PSIS could be caused by genetic deficiency in the patients who did not have any perinatal injury experience but show clear familial heredity.^3^

Previously, studies have identified several potential pathogenic genes for PSIS including *HESX1* (MIM 601802), *LHX4* (MIM 602146), *PROP1* (MIM 601538), *PROKR2* (MIM 607123), *OTX2* (MIM 600037), *SOX3* (MIM 313430), *GPR161* (MIM 612250), *POU1F1* (MIM 173110), *GLI2* (MIM 165230) and *Shh* (MIM 600725).^4^ These genes are enriched in Shh, Wnt and Notch signalling pathways, and most of them are transcription factors involved in pituitary gland development. Recently, a whole‐exome sequencing (WES) study was performed in 24 Chinese patients with isolated PSIS by Guo et al^5^, who identified several heterozygous mutations in genes associated with Notch, Shh and Wnt signalling pathways. Another study performed involving 20 isolated PSIS patients from the Netherlands suggested a non‐Mendelian polygenic aetiology of PSIS.^6^ Despite the fact that dozens of genes have been associated with PSIS, fewer than 5% of cases can be explained by known pathogenic genes, and genetic aetiology in sporadic patients is still largely undetermined. In the current study, we performed a WES study on 59 isolated patients with PSIS to identify novel germline mutations that might contribute to sporadic PSIS. The findings of our current study could extend the understanding of PSIS pathogenic genetic aetiology.

## 2 MATERIALS AND METHODS

### 2.1 Participant recruitment

A total of 59 patients who had received hormone substitution treatment in the Ruijin Hospital North, between 2016 and 2018, were recruited in the study. All patients had undergone brain MRI tests and also biochemical tests for pituitary hormone levels. PSIS was diagnosed based on the following clinical features: (a) small or absent anterior pituitary lobe, (b) interrupted or absent pituitary stalk, and (c) ectopic posterior pituitary lobe. Patients with a tumour in the brain or interrupted hypothalamic‐pituitary stalk caused by an accident were excluded. No restrictions on inheritance patterns were considered for the patients. Pituitary hormones including growth hormone (GH), gonadotropins, prolactin, cortisol, luteinizing hormone (LH), adreno‐cortico‐tropic‐hormone (ACTH), follicle‐stimulating hormone (FSH) and thyrotropin (TSH) in plasma were determined according to the clinical laboratory instructions. Each patient received a standard hormone replacement treatment according to the clinical guidelines.^7^ The study was approved by the ethics committee of Ruijin Hospital North, Shanghai Jiao Tong University School of Medicine. All participants and their legal guardians provided written informed consent.

### 2.2 Exome sequencing and bioinformatics analysis

The WES was performed using SureSelect v5 reagents (Agilent Technologies) to capture exons and the HiSeq X Ten platform (Illumina) for subsequent sequencing. Alignment was carried out with respect to the human genome assembly hg19, followed by recalibration and variant calling. Mutation sites of the genes were annotated with ANNOVAR. The gene mutations were filtered in three panels, which were constructed from the OMIM database, including the following: (a) pituitary and hypogonadotropic hypogonadism panel with 77 genes (panel 1), (b) holoprosencephaly panel with 50 genes (panel 2) and (c) midline abnormality panel with 168 genes (panel 3). Details of the panels are listed in Table S1. Then, candidate pathogenic variants were considered based on nucleotide and amino acid conservation and pathogenicity prediction by bioinformatics tools including PolyPhen‐2, SIFT, MutationTaster and CADD. We excluded the variants with population allele frequencies greater than 0.3% in the 1000 Genomes Project. Finally, variants that were recurrent in more than one patient or that were null mutations were of concern and discussed. The STRING database was used to infer the protein‐protein interactions of the identified pathogenic genes. The Sanger sequencing of both forward and reverse strands was used to further confirm the candidate pathogenic variants; the primer sequences are provided in Table S2.

## 3 RESULTS

### 3.1 Clinical characteristics of the patients

A total of 59 PSIS patients (51 men and eight women) were recruited in the present study. The mean age of this cohort was 24.03 years (range: 16‐45 years). The clinical characteristics of the patients are summarized in Table 1. Of them, 71.1% had experienced abnormal foetal position (40 with breech presentations, and two with transverse presentation), and 28.8% (17/59) had a history of temporary hypoxia during delivery. All patients had GH deficiency and LH/FSH deficiency at a post‐pubertal age, 94.9% (56/59) had TSH deficiency, and 91.5% (54/59) had ACTH deficiency.

**TABLE 1:** Clinical characteristics of the studied PSIS patients

Characteristics	PSIS patients (n = 59)
Basic information	
Gender (male/female)	51/8
Age (year)	24.03 ± 6.38
Height (cm)	
Male	162.93 ± 9.69
Female	150.69 ± 13.39
Weight (kg)	
Male	61.36 ± 14.23
Female	49.43 ± 15.16
Perinatal events	
Perinatal complication	83.1% (49/59)
Premature	13.6% (8/59)
Abnormal foetal position	71.2% (42/59)
Breech presentation	67.8% (40/59)
Transverse presentation	3.4% (2/59)
Hypoxia	28.8% (17/59)
Intracranial haemorrhage	3.4% (2/59)
Unknown	13.6% (8/59)
Normal	3.4% (2/59)
Pituitary hormone deficiency	
GH deficiency	100% (59/59)
LH/FSH deficiency	100% (59/59)
TSH deficiency	94.9% (56/59)
ACTH deficiency	91.5% (54/59)

Abbreviations: ACTH, adrenocorticotropic hormone; FSH, follicle‐stimulating hormone; GH, growth hormone; LH, luteinizing hormone; PSIS, pituitary stalk interruption syndrome; TSH, thyrotropin.

### 3.2 Main findings of whole‐exome sequencing

**FIGURE 1:**
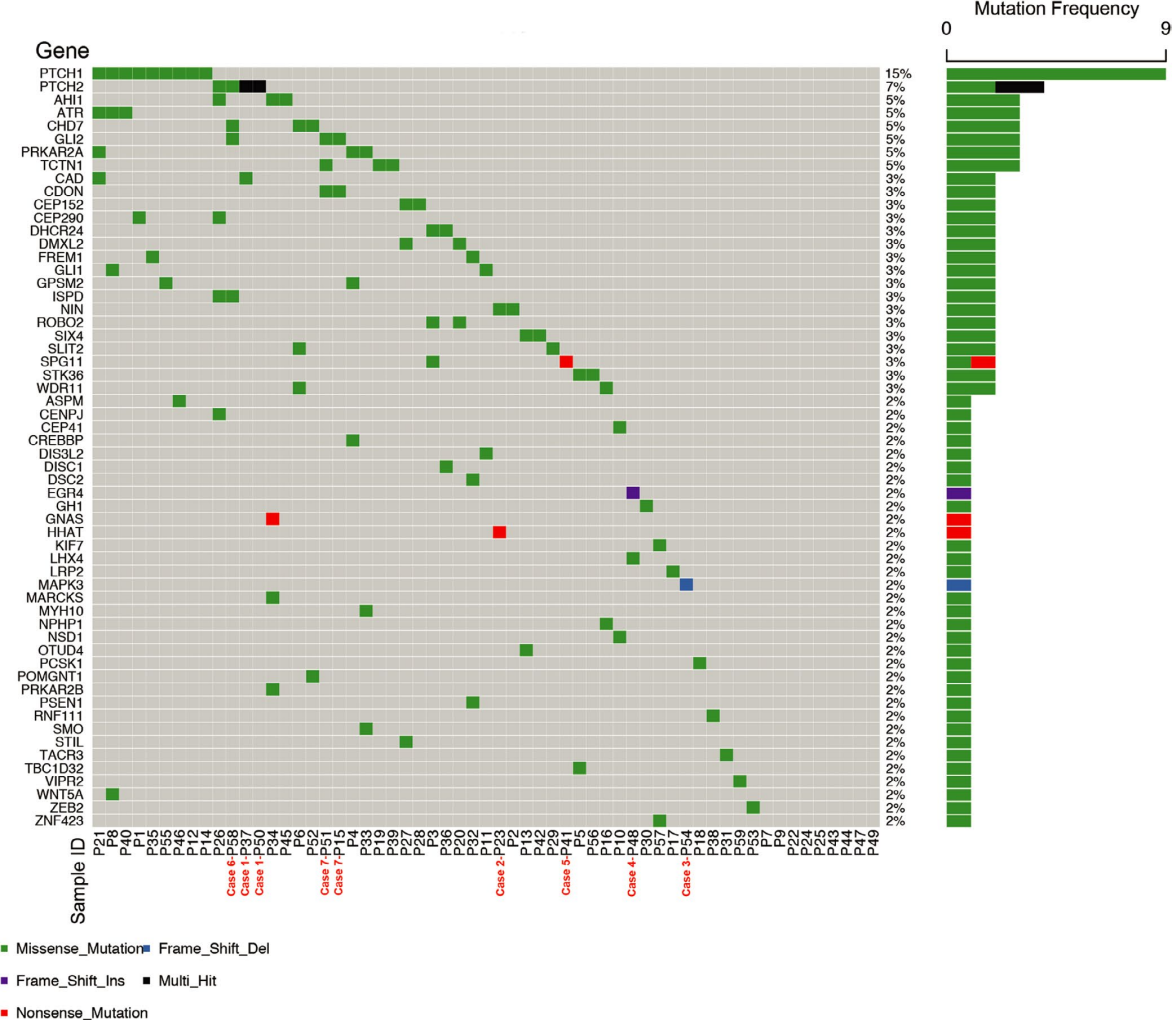
Summary of candidate pathogenic gene mutations of PSIS. The distribution of the mutations in 59 genes from 59 PSIS patients is shown

A total of 81 heterozygous variants, distributed in 59 genes, were identified in 50 patients (Figure 1, Table 2). Of them, genetic alterations in *PTCH1*, *PTCH2*, *GLI2*, *TCTN1* and *ATR* were most frequently encountered in our cohort. In addition, 37 of 59 genes showed an obvious protein‐protein interaction network as suggested by the STRING database, and 14 genes clustered into the Hedgehog pathway, including *GLI1*, *GLI2*, *PTCH1*, *PTCH2*, *CDON*, *CREBBP*, *KIF7*, *LHX4*, *HHAT*, *STK36*, *MAPK3*, *SMO*, *PRKAR2A* and *PRKAR2B* (Figure S1). Among them, *GLI2*, *PTCH2* and *PRKAR2A* had the same variant in more than two patients. Of 50 patients with potentially pathogenic variants, 31 had more than one candidate variant, suggesting a polygenic genetic aetiology of PSIS.

**TABLE 2:** Exome sequencing results of every PSIS patients including variants, in silico prediction and allele frequency in controls

Patient ID	Panel	Gene	Variant	Type	In silico prediction			Allele frequency in controls			Known phenotype
					PolyPhen‐2	MutationTaster	CADD	1000 g	Esp6500	dbSNP	
1	Panel_3	CEP290	p.R168C (c.C502T)	SNV	D	D	35	–	–	–	Joubert Syndrome
	Panel_2	PTCH1	p.A409V (c.C1226T)	SNV	P	D	27.8	0.0010	0.000077	rs2227971	Basal Cell Naevus Syndrome, Holoprosencephaly
2	Panel_3	NIN	p.E1944K (c.G5830A)	SNV	D	D	34	–	–	–	Seckel Syndrome
3	Panel_3	DHCR24	p.R444H (c.G1331A)	SNV	D	N	26.7	–	–	–	Desmosterolosis, Restrictive Dermopathy
	Panel_3	SPG11	p.L63F (c.C187T)	SNV	D	D	28	–	–	–	Spastic Paraplegia, Autosomal Recessive
	Panel_3	ROBO2	p.Y584C (c.A1751G)	SNV	D	–	23.3	0.0016	–	rs149389279	Vesicoureteral Reflux, new added PSIS candidate gene
4	Panel_3	GPSM2	p.R637W (c.C1909T)	SNV	D	D	35	0.0006	–	rs189033496	Chudley‐Mccullough Syndrome
	Panel_3	CREBBP	p.K1831R (c.A5492G)	SNV	D	D	22.9	–	–	–	Rubinstein‐Taybi Syndrome
	Panel_3	PRKAR2A	p.N344D (c.A1030G)	SNV	D	D	24.3	0.0016	–	rs117433616	Kallmann Syndrome
5	Panel_3	TBC1D32	p.K457I (c.A1370T)	SNV	D	D	29.6	–	–	–	–
	Panel_3	STK36	p.R240W (c.C718T)	SNV	D	N	34	0.0030	–	rs35038757	Congenital Hydrocephalus
6	Panel_3	SLIT2	p.R539S (c.C1615A)	SNV	D	D	34	–	–	–	Crohn's Colitis, Brain Glioma.
	Panel_1	WDR11	p.R703Q (c.G2108A)	SNV	D	D	24.8	–	–	–	Hypogonadotropic Hypogonadism
	Panel_1	CHD7	p.E2258K (c.G6772A)	SNV	D	D	27.1	–	–	–	Hypogonadotropic Hypogonadism
7	–	–	–	–	–	–	–	–	–	–	–
8	Panel_1	WNT5A	p.D375N (c.G1123A)	SNV	P	D	25.4	–	–	–	Robinow Syndrome
	Panel_3	ATR	p.I783M (c.A2349G)	SNV	D	D	19.02	–	–	–	Cutaneous Telangiectasia And Cancer Syndrome
	Panel_1	GLI1	p.A74V (c.C221T)	SNV	B	D	23.1	–	–	–	Ellis‐Van Creveld Syndrome.
	Panel_2	PTCH1	p.A1014V (c.C3041T)	SNV	D	D	32	–	–	–	Basal Cell Naevus Syndrome, Holoprosencephaly
9	–	–	–	–	–	–	–	–	–	–	–
10	Panel_3	NSD1	p.R1159Q (c.G3476A)	SNV	D	D	34	–	–	–	Sotos Syndrome, Beckwith‐Wiedemann Syndrome.
	Panel_3	CEP41	p.P10A (c.C28G)	SNV	D	D	12.92	0.0002	–	–	Joubert Syndrome
11	Panel_3	DIS3L2	p.I238V (c.A712G)	SNV	D	D	23	–	–	–	Perlman Syndrome, Wilms Tumour Predisposition
	Panel_1	GLI1	p.R557C (c.C1669T)	SNV	D	D	34	0.0006	–	rs201845227	Ellis‐Van Creveld Syndrome.
12	Panel_2	PTCH1	p.R95C (c.C283T)	SNV	P	D	25.4	0.0016	–	rs56102979	Basal Cell Naevus Syndrome, Holoprosencephaly
13	Panel_1	SIX4	p.I478S (c.T1433G)	SNV	D	D	23.5	–	–	–	Holoprosencephaly
	Panel_1	OTUD4	p.W35L (c.G104T)	SNV	D	D	34	–	–	–	PSIS, CPHD
14	Panel_2	PTCH1	p.R95C (c.C283T)	SNV	P	D	25.4	0.0016	–	rs56102979	Basal Cell Naevus Syndrome, Holoprosencephaly
15 (case 7)	Panel_1	CDON	p.V416L (c.G1246C)	SNV	B	D	17.18	0.0001	–	rs199880115	PSIS, Holoprosencephaly
		GLI2	p.Q1156E (c.C3466G)	SNV	B	D	9.074	–	–	–	PSIS, Culler‐Jones Syndrome, Holoprosencephaly
16	Panel_1	WDR11	p.Q52R (c.A155G)	SNV	D	D	24.4	0.0002	–	rs202191723	Hypogonadotropic Hypogonadism
	Panel_3	NPHP1	p.E558Q (c.G1672C)	SNV	D	D	25.7	–	–	–	Senior‐Loken Syndrome, Nephronophthisis
17	Panel_2	LRP2	p.A4148S (c.G12442T)	SNV	D	D	27.5	–	–	–	Donnai‐Barrow Syndrome, Dent Disease
18	Panel_1	PCSK1	p.V188A (c.T563C)	SNV	D	D	24.4	0.0006	–	rs183045011	Proprotein Convertase 1/3 Deficiency
19	Panel_2	TCTN1	p.S103Y (c.C308A)	SNV	D	D	22.1	0.0012	0.000171	rs117896500	Joubert Syndrome
20	Panel_1	DMXL2	p.D3015E (c.T9045G)	SNV	D	D	23.6	–	–	–	Polyendocrine‐Polyneuropathy Syndrome and Deafness
	Panel_3	ROBO2	p.Y584C (c.A1751G)	SNV	D	–	23.3	0.0016	–	rs149389279	Vesicoureteral Reflux, new added PSIS candidate gene
21	Panel_3	ATR	p.R109W (c.C325T)	SNV	D	D	27.5	0.0032	–	rs146405935	Cutaneous Telangiectasia And Cancer Syndrome
	Panel_2	CAD	p.F331I (c.T991A)	SNV	D	D	28.6	–	–	–	Epileptic Encephalopathy, Early Infantile
	Panel_3	PRKAR2A	p.N344D (c.A1030G)	SNV	D	D	24.3	0.0016	–	rs117433616	Kallmann Syndrome
	Panel_2	PTCH1	p.R827H (c.G2480A)	SNV	D	D	23.7	0.0020	–	rs138154222	Basal Cell Naevus Syndrome, Holoprosencephaly
22	–	–	–	–	–	–	–	–	–	–	–
23 (case 2)	Panel_3	NIN	p.S1965C (c.C5894G)	SNV	D	D	23	0.0006	–	rs147863467	Seckel Syndrome
	Panel_2	HHAT	p.R280X (c.C838T)	Stop‐gain	–	–	39	–	–	–	Chondrodysplasia‐Pseudohermaphroditism Syndrome
24	–	–	–	–	–	–	–	–	–	–	–
25	–	–	–	–	–	–	–	–	–	–	–
26	Panel_3	AHI1	p.K306N (c.G918T)	SNV	D	D	27.5	–	–	–	Joubert Syndrome
	Panel_3	CEP290	p.E518A (c.A1553C)	SNV	D	D	28.5	0.0006	–	rs375038986	Joubert Syndrome
	Panel_3	ISPD	p.R126C (c.C376T)	SNV	D	D	35	–	–	–	Muscular Dystrophy‐Dystroglycanopathy
	Panel_3	CENPJ	p.L3P (c.T8C)	SNV	D	D	26.4	–	–	–	Autosomal Recessive and Seckel Syndrome
	Panel_2	PTCH2	p.Q242H (c.G726C)	SNV	D	D	34	–	0.000083	rs376099036	Basal Cell Naevus Syndrome, Holoprosencephaly
27	Panel_3	CEP152	p.R1304C (c.C3910T)	SNV	D	D	25.6	–	–	–	Seckel Syndrome
	Panel_2	STIL	p.D352N (c.G1054A)	SNV	D	D	33	–	–	–	Microcephaly
	Panel_1	DMXL2	p.A381T (c.G1141A)	SNV	D	D	24.1	–	–	rs77486493	Polyendocrine‐Polyneuropathy Syndrome and Deafness,
28	Panel_3	CEP152	p.E926V (c.A2777T)	SNV	D	D	28.9	0.0010	–	rs117557829	Seckel Syndrome
29	Panel_3	SLIT2	p.S349F (c.C1046T)	SNV	D	D	33	–	–	–	Crohn's Colitis, Brain Glioma.
30	Panel_1	GH1	p.A43T (c.G127A)	SNV	D	D	29.4	–	0.000077	rs140787052	Growth Hormone Deficiency
31	Panel_1	TACR3	p.S460C (c.C1379G)	SNV	D	D	28.1	–	–	–	Hypogonadotropic Hypogonadism
32	Panel_3	DSC2	p.R833C (c.C2497T)	SNV	D	D	35	0.0020	–	rs142410803	Arrhythmogenic Right Ventricular Dysplasia
	Panel_3	PSEN1	p.L171I (c.C511A)	SNV	P	D	25.4	–	–	–	Alzheimer Disease
	Panel_3	FREM1	p.P328R (c.C983G)	SNV	D	D	23.8	–	–	–	Bifid Nose With Or Without Anorectal And Renal Anomalies
33	Panel_3	MYH10	p.R1329C (c.C3985T)	SNV	D	D	35	0.0012	–	rs370400336	Lymphangioleiomyomatosis
	Panel_3	PRKAR2A	p.N344D (c.A1030G)	SNV	D	D	24.3	0.0016	–	rs117433616	Kallmann Syndrome
	Panel_3	SMO	p.T179M (c.C536T)	SNV	D	D	24.3	0.0014	–	rs115491500	Curry‐Jones Syndrome and Basal Cell Carcinoma.
34	Panel_3	AHI1	p.R982M (c.G2945T)	SNV	D	D	26.5	–	–	–	Joubert Syndrome
	Panel_3	MARCKS	p.A202V (c.C605T)	SNV	D	N	22.3	–	–	–	Anencephaly and Bipolar Disorder.
	Panel_1	GNAS	p.G142X (c.G424T)	Stop‐gain	–	–	–	–	–	–	Mccune‐Albright Syndrome and Osseous Heteroplasia
	Panel_3	PRKAR2B	p.A310S (c.G928T)	SNV	D	D	33	0.0006	–	rs200774998	Carney Complex Variant
35	Panel_3	FREM1	p.G741S (c.G2221A)	SNV	D	D	24.7	0.0002	–	rs148111679	Bifid Nose With Or Without Anorectal And Renal Anomalies
	Panel_2	PTCH1	p.R95C (c.C283T)	SNV	P	D	25.4	0.0016	–	rs56102979	Basal Cell Naevus Syndrome, Holoprosencephaly
36	Panel_3	DHCR24	p.R462H (c.G1385A)	SNV	D	D	28.3	–	–	–	Desmosterolosis and Restrictive Dermopathy, Lethal.
	Panel_3	DISC1	p.R569W (c.C1705T)	SNV	D	D	24.7	0.0002	–	rs148111679	Schizophrenia and Schizophrenia.
37 (case 1)	Panel_2	CAD	p.G132R (c.G394A)	SNV	D	D	29.8	0.0006	–	rs145509871	Epileptic Encephalopathy, Early Infantile
	Panel_2	PTCH2	p.L104P (c.T311C)	SNV	D	D	27.7	0.0003	–	rs80168454	Basal Cell Naevus Syndrome, Holoprosencephaly
	Panel_2	PTCH2	p.S391fs (c.1172_1173del)	Frameshift	–	–	–	0.0002	–	rs56126236	Basal Cell Naevus Syndrome, Holoprosencephaly
38	Panel_3	RNF111	p.P486T (c.C1456A)	SNV	D	D	28.3	–	–	–	
39	Panel_2	TCTN1	p.A164G (c.C491G)	SNV	D	N	24.7	–	–	–	Joubert Syndrome
40	Panel_3	ATR	p.R109W (c.C325T)	SNV	D	D	27.5	0.0032	–	rs146405935	Cutaneous Telangiectasia And Cancer Syndrome
	Panel_2	PTCH1	p.R95C (c.C283T)	SNV	P	D	25.4	0.0016	–	rs56102979	Basal Cell Naevus Syndrome, Holoprosencephaly
41 (case 5)	Panel_3	SPG11	p.Q1624X (c.C4870T)	Stop‐gain	–	–	39	–	–	–	Spastic Paraplegia, Autosomal Recessive
42	Panel_1	SIX4	p.I478S (c.T1433G)	SNV	D	D	23.5	–	–	–	Pituitary Hormone Deficiency
43	–	–	–	–	–	–	–	–	–	–	–
44	–	–	–	–	–	–	–	–	–	–	–
45	Panel_3	AHI1	p.K520E (c.A1558G)	SNV	D	D	22.1	–	–	–	Joubert Syndrome
46	Panel_3	ASPM	p.L730F (c.C2188T)	SNV	D	D	25.9	–	–	–	
	Panel_2	PTCH1	p.R827H (c.G2480A)	SNV	D	D	23.7	0.0020	–	rs138154222	Basal Cell Naevus Syndrome, Holoprosencephaly
47	–	–	–	–	–	–	–	–	–	–	–
48 (case 4)	Panel_3	EGR4	p.G22fs (c.65dupG)	Frameshift	–	–	–	–	–	–	
	Panel_1	LHX4	p.P389T (c.C1165A)	SNV	B	D	16.55	0.0009	–	rs145433128	Pituitary Hormone Deficiency
49	–	–	–	–	–	–	–	–	–	–	–
50 (case 1)	Panel_2	PTCH2	p.L104P (c.T311C)	SNV	D	D	27.7	0.0003	–	rs80168454	Basal Cell Naevus Syndrome, Holoprosencephaly
	Panel_2	PTCH2	p.S391fs (c.1172_1173del)	Frameshift	–	–	–	0.0002	–	rs56126236	Basal Cell Naevus Syndrome, Holoprosencephaly
51 (case 7)	Panel_2	TCTN1	p.S103Y (c.C308A)	SNV	D	D	22.1	0.0012	0.000171	rs117896500	Joubert Syndrome
	Panel_1	CDON	p.V416L (c.G1246C)	SNV	B	D	17.18	0.0001	–	rs199880115	PSIS, Holoprosencephaly
	Panel_1	GLI2	p.Q1156E (c.C3466G)	SNV	B	D	9.074	–	–	–	PSIS, Culler‐Jones Syndrome, Holoprosencephaly
52	Panel_3	POMGNT1	p.P312S (c.C934T)	SNV	D	D	25.2	–	–	–	Muscular Dystrophy‐Dystroglycanopathy
	Panel_1	CHD7	p.E2252K (c.G6754A)	SNV	D	D	26.6	–	–	–	Hypogonadotropic Hypogonadism
53	Panel_3	ZEB2	p.L1014I (c.C3040A)	SNV	D	D	29.3	–	–	–	Mowat‐Wilson Syndrome and Mowat‐Wilson Syndrome
54 (case 3)	Panel_3	MAPK3	p.H50fs (c.150_153del)	Frameshift	–	–	–	–	–	–	autism and neutrophil migration
55	Panel_3	GPSM2	p.R637W (c.C1909T)	SNV	D	D	35	0.0006	–	rs189033496	Chudley‐Mccullough Syndrome
	Panel_2	PTCH1	p.R95C (c.C283T)	SNV	P	D	25.4	0.0016	–	rs56102979	Basal Cell Naevus Syndrome, Holoprosencephaly
56	Panel_3	STK36	p.R240W (c.C718T)	SNV	D	N	34	0.0030	–	rs35038757	Congenital Hydrocephalus
57	Panel_3	KIF7	p.T807M (c.C2420T)	SNV	D	D	29.8	–	–	–	Al‐Gazali‐Bakalinova Syndrome and Acrocallosal Syndrome.
	Panel_3	ZNF423	p.G453S (c.G1357A)	SNV	D	D	13.36	0.0010	–	rs201929999	Joubert Syndrome With Oculorenal Anomalies
58 (case 6)	Panel_1	CHD7	p.P394S (c.C1180T)	SNV	D	D	20.1	0.0002	–	rs182061582	Hypogonadotropic Hypogonadism
	Panel_3	ISPD	p.R116H (c.G347A)	SNV	D	N	24	–	–	–	Muscular Dystrophy‐Dystroglycanopathy
	Panel_1	GLI2	p.A524T (c.G1570A)	SNV	D	D	17.78	–	–	–	PSIS, Culler‐Jones Syndrome, Holoprosencephaly
	Panel_2	PTCH2	p.S263N (c.G788A)	SNV	B	D	23.3	–	–	rs77102909	Basal Cell Naevus Syndrome, Holoprosencephaly
59	Panel_2	VIPR2	p.R2W (c.C4T)	SNV	D	N	24.4	–	–	–	Holoprosencephaly

Note

Seveb Patients with compound mutations in Hedgehog pathway: P4 with CREBBP and PRKAR2A, P8 with GLI1 and PTCH1, P15 with CDON and GLI2, P21 with PRKAR2A and PTCH1, P33 with PRKAR2A and SMO, P51 with CDON and GLI2 and P58 with GLI2 and PTCH2.

Abbreviations: B, benign; D, damage; P, possible damage; SNV, missense mutation due to single nuclear polymorphisms.

In panel 1 associated with pituitary and hypogonadotropic hypogonadism, we found that candidate pathogenic variants were present in *WDR11*, *CHD7*, *WNT5A*, *GLI*, *SIX4*, *OTUD4*, *CDON*, *PCSK1*, *DMXL2*, *GH1*, *TACR3*, *GNAS*, *SIX4* and *LHX4*. GLI2 and SIX4 had the same variants distributed in two patients, and GNAS had a stop‐gain mutation (Table 2). In panel 2 associated with holoprosencephaly malformation, candidate pathogenic variants were present in *PTCH1/2*, *LRP2*, *TCTN1*, *CAD*, *HHAT*, *STIL* and *VIPR2* (Table 2). Of them, *PTCH1/2* and *TCTN1* had the same variants in more than one patients and HHAT had a nonsense mutation. Mutations in *PTCH1* and *PTCH2* were the most frequent, with an overall incidence of 22% (13/59). Four missense variants in *PTCH1* and three missense and one frameshift variants in *PTCH2* were identified in 13 patients (Table S3). The frameshift variant of *PTCH2* is a known pathogenic variant of basal cell naevus syndrome. In panel 3 associated with midline abnormality, recurrent candidate pathogenic variants were present in *ROBO2*, *GPSM2*, *ATR* and *PRKAR2A*. Frameshift mutations were found in *MAPK3*, and nonsense mutations were found in *EGR4* and SPG11 (Table 2).

### 3.3 Novel pathogenic genes associated with PSIS

Well‐documented pathogenic variants of PSIS (*HESX1*, *LHX4*, *PROP1*, *PROKR2*, *OTX2*, *CDON*, *SOX3*, *GPR161*, *POU1F1*, *GLI1*, *GLI2*, *OTUD4*, *ROBO2* and *Shh*) based on the ClinVar tool were not found, and only several rare, candidate pathogenic variants were found in *GLI1*, *GLI2*, *LHX4*, *CDON*, *ROBO2* and *OTUD4*. These variants were suggested to be damaging based on in silico prediction and low allele frequencies, but the interpretation of these variants was classified as unknown significance by ClinVar. Among 59 genes, the variants that led to truncation of the protein or de novo mutations, forming homozygous or compound heterozygous variants, were considered pathogenic and discussed in detail as follows (Table 3). These were interpreted as pathogenic genes or likely pathogenic genes according to the recommendation of the American College of Medical Genetics and Genomics.^8^

**TABLE 3:** Frequency and pathogenicity classification of pathogenic variants and likely pathogenic variants

Panel	Patient ID	Gene	Variant	Type	Inherent	In silico prediction			Allele frequency in controls			Evidence of pathogenic
						PolyPhen‐2	MutationTaster	CADD	1000 g	Esp6500	dbSNP	
Panel_2	P37,P50	PTCH2	p.S391fs (c.1172_1173del)	Frameshift	Paternal	–	–	–	0.0002	–	rs56126236	Pathogenic (PVS1 + PS1+PM2)
Panel_2		PTCH2	p.L104P (c.T311C)	Missense	Maternal	0.995	1,D	27.7	0.0003	–	rs80168454	
Panel_2	P23	HHAT	p.R280X (c.C838T)	Stop‐gain	De novo	–	–	39	–	–	–	Pathogenic (PVS1 + PS2+PM2)
Panel_3	P54	MAPK3	p.H50fs (c.150_153del)	Frameshift	Maternal	–	–	–	–	–	–	L Pathogenic (PVS1 + PM2+PP3)
Panel_3	P48	EGR4	p.G22fs (c.65dupG)	Frameshift	Maternal	–	–	–	–	–	–	L Pathogenic (PVS1 + PM2+PP3)
Panel_1		LHX4	p.P389T (c.C1165A)	Missense	Paternal	0.411	0.999,D	16.55	0.0009	–	rs145433128	
Panel_3	P41	SPG11	p.Q1624X (c.C4870T)	Stop‐gain	Maternal	–	–	39	–	–	–	L Pathogenic (PVS1 + PM2+PP3)
Panel_1	P58	GLI2	p.A524T (c.G1570A)	Missense	Maternal	1	1,D	17.78	–	–	–	L Pathogenic (PM1 + PM2+PP3)
Panel_2		PTCH2	p.S263N (c.G788A)	Missense	Paternal	0.43	0.932,D	23.3	–	–	rs77102909	
Panel_1	P15,P51	CDON	p.V416L (c.G1246C)	Missense	Maternal	0.164	0.994,D	17.18	0.0001	–	rs199880115	L Pathogenic (PM2 + PP3)
Panel_1		GLI2	p.Q1156E (c.C3466G)	Missense	Paternal	0.103	1,D	9.074	–	–	–	

Note

Panel_1: Hypogonadotropic Hypogonadism Panel; Panel_2: Holoprosencephaly Panel; Panel_3: Midline abnormally Panel; L Pathogenic: likely Pathogenic.

PVS1: null variant (nonsense, frameshift) in a gene where LOF is a known mechanism of disease.

PS1: Same amino acid change as a previously established pathogenic variant regardless of nucleotide change.

PS2: De novo (both maternity and paternity confirmed) in a patient with the disease and no family history.

PM1: Mutation in well‐established functional domain without benign variation.

PM2: Absent from controls (or at extremely low frequency if recessive) in Exome Sequencing Project, 1000 Genomes Project or Exome Aggregation Consortium.

PP3: Mutation with multiple lines of computational evidence supports a deleterious effect on the gene or gene product.

### 3.4 Case 1. Frameshift variant of *PTCH2* (P37 and P50)

**FIGURE 2:**
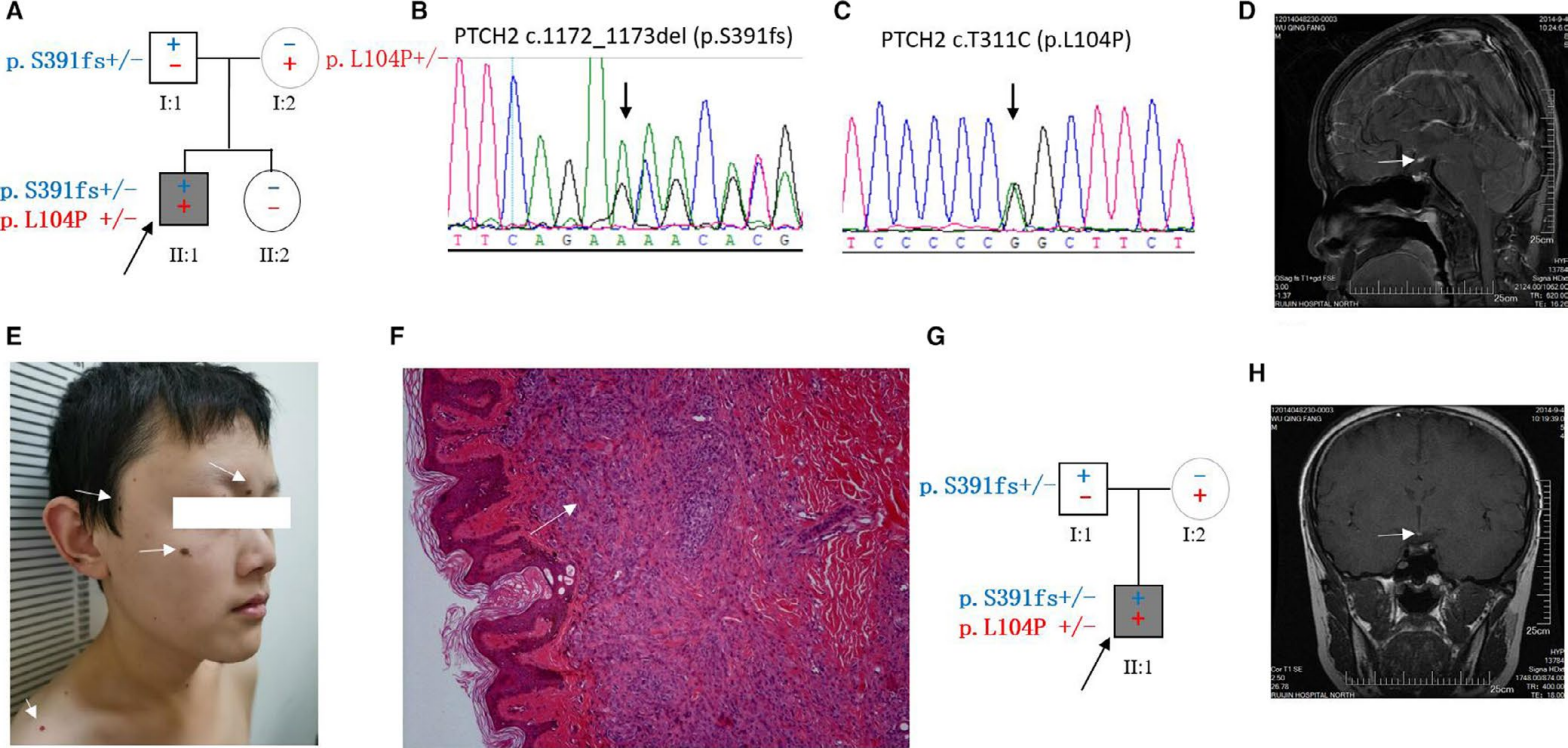
PTCH2 biallelic heterozygous variants. A, The pedigrees of patient P50. B‐C, The corresponding chromatograms of patient P50. D, The MRI image of P50, conform typical PSIS. E, Multiple congenital pigmented naevi in body of P50. F, Pathological examination shows intradermal naevus, exclude the possibility of Gorlin syndrome. G, The pedigrees of P37. H, The MRI image of P37, conform typical PSIS

P37 was a 27‐year‐old man (Figure 2A‐2F), whereas P50 was a 24‐year‐old man (Figure 2G‐2H). They had no genetic relationship. They had disclosed pituitary hypoplasia and combined pituitary hormone deficiency (CPHD). Both were found to harbour a paternal frameshift variant of *PTCH2* (c.1172_1173del, p.S391fs) and the same maternal missense mutation in *PTCH2* (c.T311C, p.L104P). p.S391fs was interpreted as a pathogenic gene with evidence of PVS1 (null variant [nonsense, frameshift] in a gene), PS1 (same amino acid change as a previously established pathogenic variant in Gorlin syndrome) and PM2 (absent from controls). As *PTCH2* is a well‐known pathogenic gene of Gorlin syndrome, P37 and P50 received careful physical examination and pathological biopsy to exclude this possibility. Although P50 had multiple congenital pigmented naevi in skin of the face and back, pathological examination excluded the possibility of basal cell naevus syndrome, with the diagnosis of intradermal naevus. P50 had a sister with wild‐type *PTCH2*, who was asymptomatic. P37 described himself as having diabetes insipidus in childhood and recovered after treatment. His physical examination did not show any abnormalities.

### 3.5 Case 2. De novo variant of *HHAT* (P23)

**FIGURE 3:**
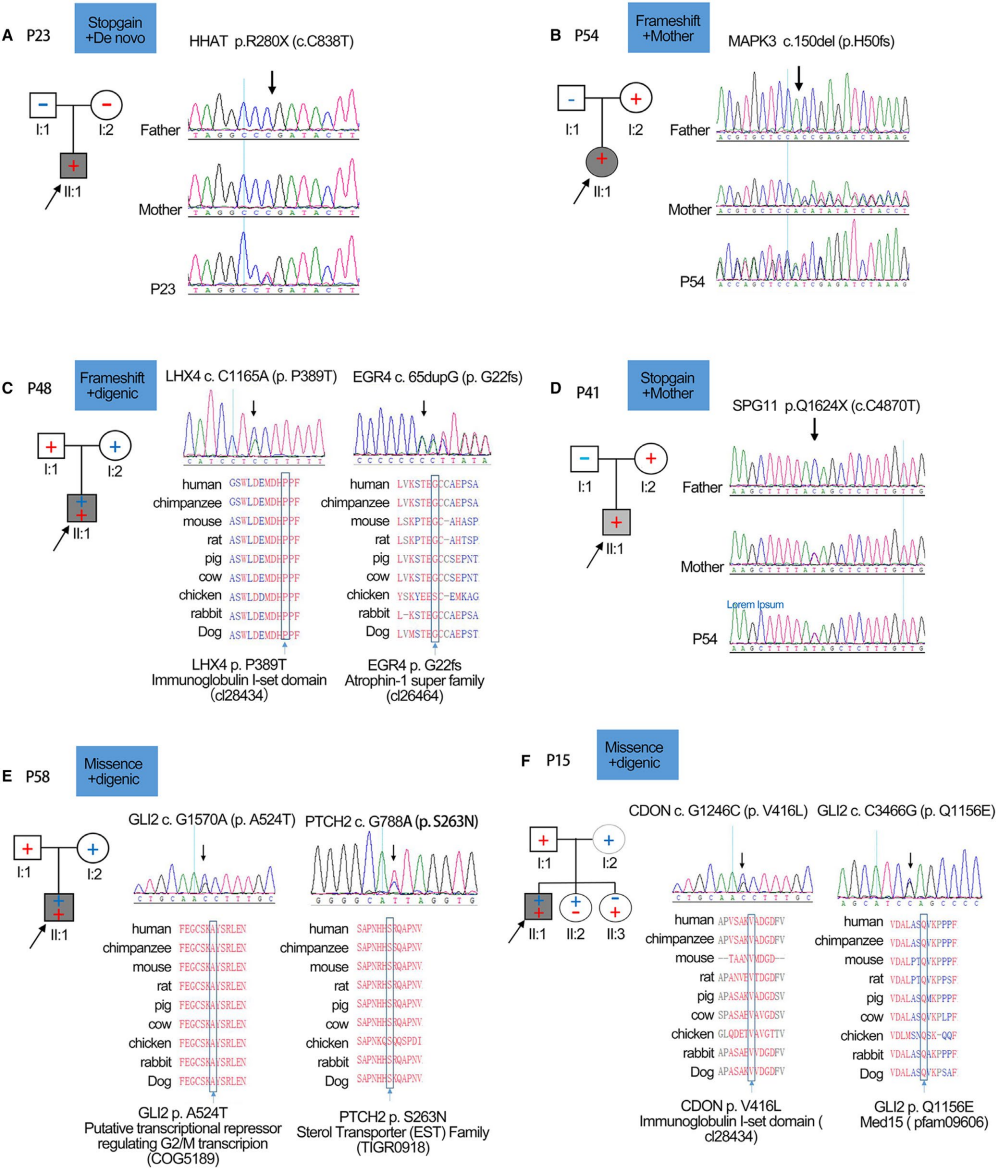
The family pedigrees of probands with a diagnosis of PSIS. The pedigrees are shown in the top left, the corresponding chromatograms are shown in the top right, and missense mutations located in the highly conserved region of proteins are shown in the bottom. A, HHAT p.R280X (c.C838T), a stop‐gain and de novo variant, identified in affected proband P23, but not his parents. B, MAPK3 p.H50fs (c.150_153del), a frameshift mutation, identified in P54 and his mother. C, EGR4 p.G22fs (c.65dupG), a frameshift mutation, identified in P48 and his mother, whereas LHX4 p.P389T (c.C1165A) identified in affected members and his father. D, SPG11 p.Q1624X (c.C4870T), a stop‐gain mutation, identified in P41 and his mother. E, GLI2 p.A524T (c.G1570A), identified in P58 and his mother, whereas PTCH2 p.S263N (c.G788A) identified in affected members and his father. F, CDON p.V416L (c.G1246C) identified in P15 and his father, whereas GLI2 p.Q1156E (c.C3466G) was derived from the mother

P23 was a 21‐year‐old man with short stature and CPHD (GH, TSH, ACTH and gonadotropin deficiency; Figure 3A). He had the perinatal complication in which feet appear first. The patient harboured a stop‐gain mutation in *HHAT* (c.C838T, p.R280X), a de novo mutation that was not detected in his parents. p.R280X was interpreted as a pathogenic gene with evidence of PVS1 (null variant), PS2 (de novo) and PM2 (absent from controls). HHAT is a hedgehog acyltransferase, and diseases associated with HHAT include chondrodysplasia‐pseudohermaphroditism syndrome and ancylostomiasis. However, P23 did not have clinical phenotypes of these diseases. Besides the *HHAT* nonsense mutation, P23 also had a maternal missense mutation in *NIN* (c.C5894G, p.S1965C).

### 3.6 Case 3. Frameshift variant of *MAPK3* (P54)

P54 was an 18‐year‐old female who experienced hypoxic coma for 2 days due to amniotic fluid aspiration after caesarean delivery (Figure 3B). She had some special developmental malformations and mental retardation with right eye strabismus and visual impairment. Her visual acuity was 0.15 in the right eye and 0.5 in the left eye. She could not walk until she was 2 years old. She could hardly concentrate and was a poor learner. A 2‐bp deletion in *MAPK3* at nucleotide 150 (c.150_153del) was found, which resulted in premature termination of the protein p.H50fs. MAPK3 p.H50fs was interpreted as a likely pathogenic gene with evidence of PVS1 (null variant), PM2 (absent from controls) and PP3 (damaging based on in silico prediction). MAPK3 is associated with autism and neutrophil migration. For P54, the possibility of autism was ruled out. Her father had the same mutation, although he was asymptomatic.

### 3.7 Case 4. Compound heterozygous variants including *EGR4* frameshift and *LHX4* (P48)

P48 was a 23‐year‐old man, with short stature and CPHD (Figure 3C). He had the perinatal complication of abnormal foetal position (breech delivery). A frameshift deletion, c.65dupG (p.G22fs), was detected in P48, inherited from his mother. *EGR4* p.G22fs was interpreted as a likely pathogenic gene with evidence of PVS1 (null variant), PM2 (absent from controls) and PP3 (damaging based on in silico prediction). In addition, he had a missense variant of *LHX4* (c.G788A, p.S263N) from his father. Although LHX4 is a well‐documented PSIS gene, the clinical significance of this variant is unknown.

### 3.8 Case 5. Nonsense variant of *SPG11* (P41)

P41 was a 19‐year‐old man, with short stature, CPHD (GH, TSH, ACTH and gonadotropin deficiency) and typical MRI characteristics of PSIS (Figure 3D). He had perinatal complications with breech delivery and a history of hypoxia at birth. A stop‐gain mutation in *SPG11* (c.C4870T, p.Q1624X) was found, which was inherited from his mother. *SPG11* p.Q1624X was interpreted as a likely pathogenic gene with evidence of PVS1 (null variant), PM2 (absent from controls) and PP3 (damaging based on in silico prediction). His mother, who had the same mutation, was asymptomatic.

### 3.9 Case 6. Compound heterozygous variants of *GLI2* and *PTCH2* (P58)

P58 was a 28‐year‐old man. He had perinatal injury (feet appear first and history of hypoxia at birth). We found a missense mutation in *GLI2* (c.G1570A, p.A524T) inherited from his mother (Figure 3E). GLI2 c.G1570A occurred in the putative transcriptional repressor domain involved in regulating G2/M transcription, which might severely affect the development of pituitary cells. PTCH2 (c.G788A, p.S263N), inherited from his father, occurred in the sterol transporter family domain. The mutation was classified as likely pathogenic with evidence of PM1 (mutation in well‐established functional domain), PM2 (extremely low frequency) and PP3 (damage in silico prediction).

### 3.10 Case 7. Compound heterozygous variants of *CDON* and *GLI2* (P15 and P51)

P15 was a 20‐year‐old man, whereas P51 was a 22‐year‐old man. P15 had perinatal complications (feet appear first) and short stature, along with CPHD and typical MRI characteristics (Figure 3F). Both P15 and P51 were detected with compound heterozygous variants of *CDON* (c.G1246C, p.V416L) from their mothers and *GLI2* (c.C3466G, p.Q1156E) from their fathers. Both *CDON* p.V416L and *GLI2* p.Q1156E are very rare (minor allele frequency [MAF] of 0.0001 and 0.0000, respectively) and were predicted to be possibly damaging by MutationTaster. P15 had two sisters with a normal phenotype, and the possibility of combined mutations in *CDON* and *GLI2* was excluded by genetically test.

## 4 DISCUSSION

In the present study, based on WES of 59 isolated patients, we identified five novel candidate pathogenic genes for PSIS, including *PTCH2*, *HHAT*, *MAPK3*, *EGR4* and *SPG11* (Table 3), as well as six candidate pathogenic variants in the documented PSIS genes of *GLI1*, *GLI2*, *ROBO2*, *OTUD4*, *LHX4* and *CDON* (Table 2). The most frequent mutations were found in *PTCH1*, *PTCH2*, *GLI2*, *TCTN1* and *ATR*, whereas null mutations were found in *PTCH2*, *HHAT*, *MAPK3*, *EGR4* and *GNAS*. Most variants from the target panels were inherited from an unaffected parent, except for *HHAT*, which was a de novo mutation in PSIS patient P23.

The most frequent mutations and genes contain null mutations were concerned. *TCTN1* (tectonic family member 1) encodes a secreted and transmembrane protein. The orthologous gene in mice modulates hedgehog signal transduction downstream of smoothened (Smo) and rab23.^9^ Therefore, the association between *TCTN1* and PSIS might be related to activation or inhibition of the hedgehog pathway. *ROBO2* has been reported as a novel candidate PSIS gene in two independent studies. Bashamboo et al^10^ found heterozygous frameshift, nonsense and missense mutations in *ROBO1* in two familial cases. Zwaveling et al^6^ identified *ROBO2* as a new candidate gene for isolated PSIS. GNAS has a highly complex imprinted expression pattern, including four alternative promoters and 5′ exons, as well as the alternative splicing of downstream exons. Considering that multiple transcript variants encoding different isoforms have been found without specific phenotypes, this nonsense variant of *GNAS* was ruled out from pathogenic variants of PSIS. Further, well‐documented PSIS pathogenic genes, such as *HESX1*, *LHX4 and GLI2*, are likely related to incomplete or reduced penetrance, which might contribute to the genetic background of disease development.^11^

### 4.1 Mutations in the Hedgehog signalling pathway

Fourteen of the 59 mutation genes were enriched in the Hedgehog signalling pathway (*GLI1*, *GLI2*, *PTCH1*, *PTCH2*, *CDON*, *CREBBP*, *KIF7*, *LHX4*, *HHAT*, *STK36*, *MAPK3*, *SMO*, *PRKAR2A* and *PRKAR2B*), which indicated that abnormal Hedgehog signalling might lead to a PSIS phenotype. We noticed that seven patients carried two compound mutations in the Hedgehog pathway (Table 2). GLI1 and GLI2 are transcription factors downstream of the Hedgehog signalling pathway, which are involved in early ventral forebrain and pituitary development. They are most frequently mutated in patients with holoprosencephaly and pituitary abnormalities.^12^, ^13^ In mouse models, the inactivation of GLI2 leads to absence of the pituitary and an abnormal midline central diencephalon; homozygous deletion of both GLI1 and GLI2 results in complete absence of the pituitary.^14^ According to our study, two missense variants in *GLI1* (c.C1669T:p.R557C; c.C221T:p.A74V) and three missense variants in *GLI2* (c.G376A:p.A126T; c.G2554A:p.A852T; c.C4450G:p.Q1484E) were found with a MAF < 0.3%. The overall prevalence of *GLI* mutations was 10.2% (6/59). P58 (case 6) had a compound variant of *GLI2* (from the maternal side) and *PTCH2* (from the paternal side). Both mutations were predicted to be possibly damaging by the MutationTaster algorithm. Especially, the GLI2 c.G1570A mutation occurred in the putative transcriptional repressor domain regulating G2/M transcription, which might severely affect the development of pituitary cells.

### 4.2 Novel pathogenic variants

#### 4.2.1 PTCH2

PTCH1 and PTCH2 are negative‐feedback regulators of Hedgehog signal transduction that function by targeting the transmembrane molecule Smoothened. Therefore, loss‐of‐function mutations in PTCH1/2 might lead to activation of the Hedgehog signalling.^15^, ^16^ Previously, studies suggested that both *PTCH1* and *PTCH2* are causative genes of Gorlin syndrome and holoprosencephaly.^17^, ^18^ *PTCH2* p.S391fs was found in a 13‐year‐old Japanese girl with basal cell naevus syndrome (BCNS; 109400) who did not have a mutation in the *PTCH1* or *SUFU* gene.^16^ In P37 and P50 (Figure 2), we found two sporadic families with the p.S391fs mutation from the paternal side, combined with a p.L104P missense mutation from the maternal side. P50 had combined symptoms of multiple congenital pigmented naevi, whereas pathology showed intradermal naevus, excluding the possibility of Gorlin syndrome. Further, PTCH1, PTCH2 and HHIP1 collectively govern the ligand‐dependent feedback inhibition of vertebrate Shh signalling, which restricts constitutive Shh pathway activation in the developing nervous system.^19^ Constitutive Shh signal activation has a close relationship with PSIS or CPHD; thus, Shh, GLI2 and CDON have been successively reported as PSIS candidate genes.^20^ P37 and P50 had a biallelic frameshift heterozygous mutation in *PTCH2*, suggesting that *PTCH2* might be the novel pathogenic gene of PSIS.

#### 4.2.2 HHAT

HHAT is a hedgehog acyltransferase, required for the post‐translational palmitoylation of Hedgehog proteins. Abdel‐Salam et al^21^ reported a biallelic novel missense *HHAT* variant that might cause syndromic microcephaly and cerebellar‐vermis hypoplasia. *HHAT* mutations can also be indicative of severe acrania‐holoprosencephaly‐agnathia craniofacial defects. Loss‐of‐function HHAT in mouse models leads to holoprosencephaly, which mimics the severe condition observed in humans.^22^ Previous studies have provided clinical evidence for the essential roles of HHAT in human testicular organogenesis and embryonic development. PSIS was suggested as a mild form of holoprosencephaly,^23^ and P23 had a de novo stop‐gain mutation in *HHAT* (Figure 3A). It is highly possible that *HHAT* p.R280X is a novel pathogenic gene of PSIS.

#### 4.2.3 MAPK3

Recurrent *MAPK3* missense mutations have been found in neurodevelopmental diseases, such as ASD, ID and NDDs.^24^ MAPK3 is a key regulator of the syndrome involved in axon targeting and the regulation of cortical cytoarchitecture.^25^ Besides pituitary hormone deficiency, P54 (Figure 3B) actually had certain aspects of mental retardation, presenting with problems in understanding and lacking the ability of comprehensive memory and language expression. Her mother also had the same mutation, although she was asymptomatic for PSIS. The patient had definite hypoxia due to amniotic fluid aspiration. This would act as an environmental exposure, which promotes dominance of the MAPK3 frameshift mutation. We suspected the *MAPK3* p.H50fs mutation to be a novel PSIS pathogenic gene with a wide range of midline abnormalities; however, this needs to be confirmed by more studies.

#### 4.2.4 EGR4

Early growth response protein (EGR4) is a transcriptional regulator that is required for mitogenesis and differentiation. EGR4 has been reported to participate in fertility development during the regulation of LH secretion or posterior hindbrain development.^26^ Consistent with the EGR4 function in fertility, P48 (Figure 3C) showed poor responses to HCG (human chorionic gonadotropin) substitution therapy. Substitution therapy was initiated with levothyroxine and hydrocortisone, and delayed puberty was treated with HCG. After more than 1 year of treatment with HCG, the patient still had lower LH and FSH levels. Although the testicles became larger, the patient still had azoospermia, as suggested by a sperm test.

#### 4.2.5 SPG11

SPG11 is a transmembrane protein that is phosphorylated upon DNA damage. Mutations in SPG11 comprise a major cause of spastic paraplegia with a thin corpus callosum.^27^ It is expressed ubiquitously in the nervous system but most prominently in the cerebellum, cerebral cortex, hippocampus and pineal gland. Loss‐of‐function SPG11 was identified in hereditary spastic paraplegia patients.^28^ P41 (Figure 3D) had a *SPG11* nonsense mutation in c.C4870T, which was absent in the control population. For P41, the possibility of spastic paraplegia was excluded, and we suspected that the *SPG11* p.Q1624X mutation is a novel PSIS pathogenic gene involved in nervous system development.

### 4.3 Perinatal adverse events

Perinatal adverse events, including dystocia (83.1%, 49/59), abnormal foetal position (71.2%, 42/59) and history of hypoxia (28.8%, 17/59), were found to be more frequent in the PSIS patients in the current study (Table 1), which is consistent with results of another study performed by Zheng et al^29^ wherein the prevalence of perinatal complications was 100%. Another study performed on Chinese PSIS patients showed that breech delivery occurred in 88.9% patients and a history of dystocia was noted for 34.5% patients.^30^ These results suggested the close relationship between breech delivery and PSIS patients. For the current study, many PSIS patients came from relatively underdeveloped rural areas and regular prenatal examinations had not yet been established at the time of birth, which might have led to a higher incidence of perinatal complications. A relatively lower incidence of breech delivery (18%‐20.7%) and neonatal distress (20.6%‐26%) was noted in the European PSIS population^31^; however, the perinatal injury rate was much higher than that in the general population. These results demonstrated the roles of perinatal injury in PSIS aetiology.

In conclusion, the exome sequencing analysis of PSIS patients identified 81 germline mutations in 50 patients, and gene mutations in *PTCH2*, *HHAT*, *MAPK3*, *EGR4*, *SPG11*, *GLI2* and *CDON* could be potential pathogenic candidates in Chinese PSIS patients. Genes involved in the Hedgehog signalling pathways play critical roles in the PSIS development. However, these need to be confirmed with more studies.

## CONFLICT OF INTEREST

There is no conflict of interest that could be perceived as prejudicing the impartiality of the research reported.

## AUTHOR CONTRIBUTIONS

**Xuqian Fangxuqian:** Conceptualization (lead); Data curation (lead); Formal analysis (lead); Writing‐original draft (lead). **Yuwen Zhang:** Data curation (equal); Investigation (equal); Resources (equal). **Jialin Cai:** Investigation (equal); Visualization (equal). **Tingwei Lu:** Formal analysis (equal); Software (equal). **Junjie Hu:** Funding acquisition (equal); Supervision (equal); Writing‐review & editing (equal). **Fei Yuan:** Methodology (equal); Supervision (equal); Writing‐review & editing (equal). **Peizhan Chen:** Conceptualization (equal); Funding acquisition (equal); Methodology (equal); Resources (equal); Supervision (equal); Visualization (equal); Writing‐review & editing (equal).

## Supporting information

Fig S1

Table S1

Table S2

Table S3

## Data Availability

All data generated or analysed during this study are included in this article.
