# Supplementary figures and images for: Identification of novel candidate pathogenic genes in pituitary stalk interruption syndrome by whole‐exome sequencing
Source: J Cell Mol Med. 2020 Aug 31;24(20):11703–17. doi: 10.1111/jcmm.15781 (PMC7579688; doi:10.1111/jcmm.15781)

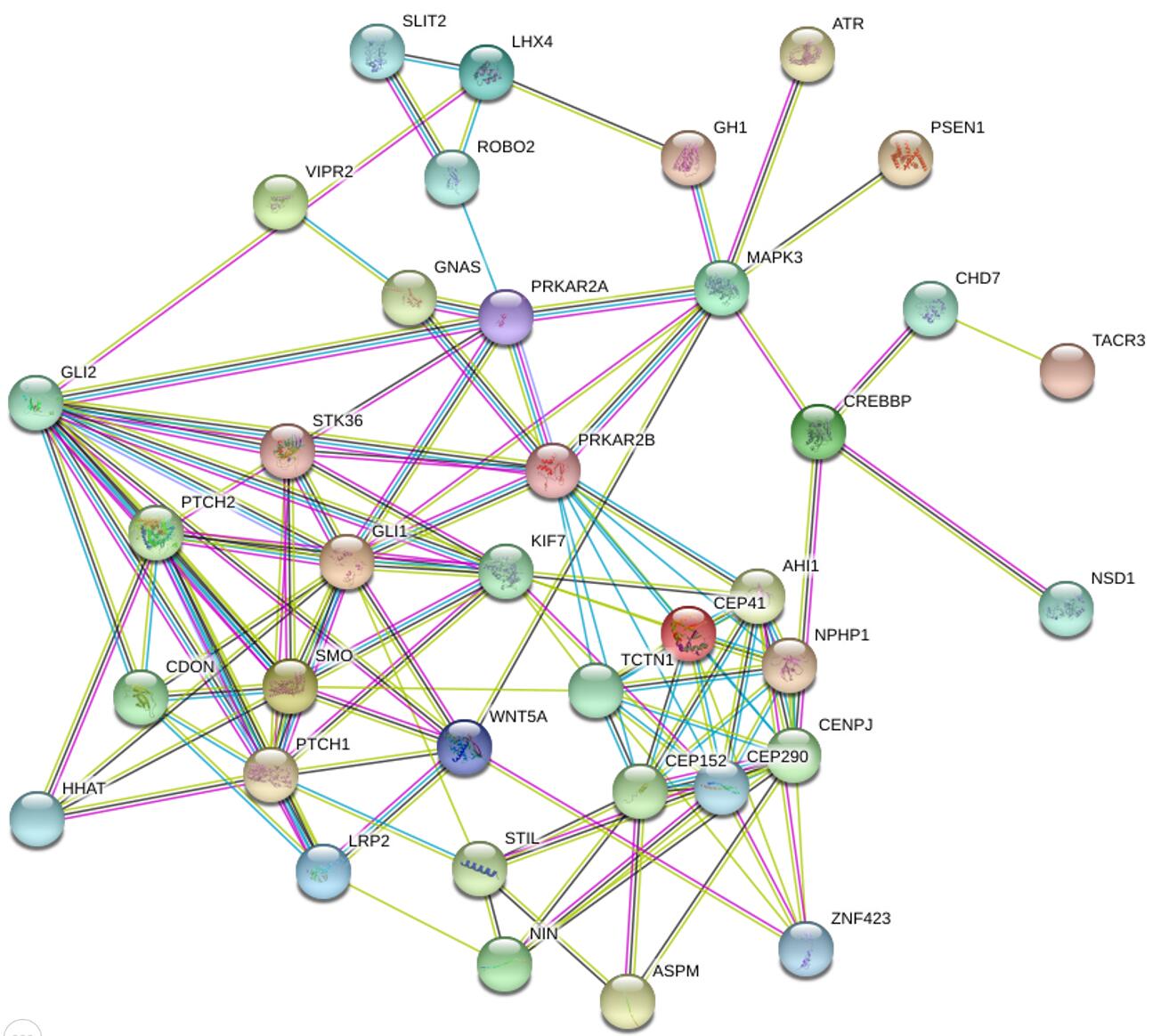

Supplement: Supplementary file 1 — Fig S1 [file JCMM-24-11703-s001.jpg]
