# Supplementary material for: Identification of novel candidate pathogenic genes in pituitary stalk interruption syndrome by whole‐exome sequencing
Source: J Cell Mol Med. 2020 Aug 31;24(20):11703–17. doi: 10.1111/jcmm.15781 (PMC7579688; doi:10.1111/jcmm.15781)
Supplement: Supplementary file 2 — Table S1 [file JCMM-24-11703-s002.docx]

Supplementary Table 1：Target Searching Panels

| 1 pituitary and hypogonadotropic hypogonadism Panel（n=77） |
| --- |
| ARNT2，AXL，BMP2，BMP4，CCDC141，CDON，CHD7，DMXL2，FEZF1，FGF10，FGF17，FGF18，FGF8，FGFR1，GATA2，GH1，GHRH，GHRHR，GLI1，GLI2，GLI3，GLI4，GLI5，GLI6，GNAS，GNRH1，GNRHR，GPR161，HESX1，HS6ST1，IGSF1，KAL1，KISS1，KISS1R，LEP，LEPR，LHX3，LHX4，NR0B1，NR5A1，NSMF，OL14RD，OTUD4，OTX2，PAX6，PCSK1，PITX1，PITX2，PNPLA6，POMC，POU1F1，PROK2，PROKR2，PROP1，RNF216，SEMA3A，SEMA3E，SEMA7A，SHH，SIX1，SIX2，SIX3，SIX4，SIX5，SIX6，SOX1，SOX10，SOX2，SOX3，STS，TAC3，TACR3，TBX19，TGIF，THRB，WDR11，WNT5a |
| 2 holoprosencephaly Panel（n=50） |
| AMER1，ARX，B9D1，BMP1，CAD，CASP2，CC2D2A，CDC42，CDON，DHCR7，DISP1，DKK1，DLL1，EAPP，EMID2，EMX2，FAT1，FGF8，FGFR1，FOXH1，FUZ，GAS1，GDF1，HHAT，INTU，KATNB1，LRP2，MNX1，NODAL，NOG，NOSIP，PPP2CA，PPP2CB，PTCH1，RHEB，RUNX2，SHH，SIX3，STIL，SUFU，TCTN1，TDGF1，TGIF，TGIF1，TMEM1，TTBK2，VIPR2，ZFP161，ZIC2，PTCH2 |
|  |
| 3 midline abnormality Panel（n=168） |
| MARCKS，BMP4，SKI，TGFB3，RAG1，ZIC3，SIM2，GSK3B，ALX3，HDCPH1，SOX3，IFT172，NR2F1，CEP290，CUL4B，PKD2，CDH1，PTCH1，DSC2，TFAP2A，NYS3，KIF3A，THAS，FGFR1，PACD，ROBO2，MID1，MSX2，PSEN1，RNF111，ADH1A，AHI1，PRRX1，OFC14，ROR2，GLI3，CHD7，SOX9，COL2A1，MECP2，NKX2-5，MYH10，PITX2，HESX1，TMEM67，SLIT2，HPE1，OTX2，ACTB，FGF8，OSR2，EXT1，RPGRIP1L，STIL，SALL4，H19，DISTAL，TBC1D32，ZEB2，TGIF，SMAD6，ZNF423，FREM1，ZIC2，FOXG1，BMP2，UNC5C，HEY2，OFD1，PKD1L1，VANGL2，VHL，CDON，TMEM216，GLI2，SATB2，GLA，APOA1，AKT3，AMPD2，ANOP1，ARID1B，ARL13B，ARX，ASPM，ATR，ATRX，B9D1，B9D2，BCOR，C12ORF57，CASK，CC2D2A，CENPJ，CEP152，CEP41，CEP63，CREBBP，CTBP1，AS1，DCX，DHCR7，DHCR24，DIS3L2，DISC1，EFNB1，EOMES，EP300，EPG5，FGFR2，FH，FKRP，FKTN，FLNA，GPSM2，GTDC2，HCCS，HS6ST1，HYLS1，IGBP1，IGF1，INPP5E，ISPD，KAT6B，KCC3，KIF7，L1CAM，LARGE，LRP2，MED12，MKS1，NDE1，NFIX，NIN，NPHP1，NPHP3，NSD1，OTX1，PAX6，PDHA1，PDHB，POMGNT1，POMT1，POMT2，PYCR1，RAB18，RAB3GAP1，RAB3GAP2，RBBP8，RBM10，RELN，RNU4ATAC，RPS6KA3，SCKL3，SOX2，SPG11，STRA6，TCF4，TCTN1，TCTN2，TCTN3，TMEM138，TMEM237，TUBA1A，TUBB2B，TUBB3，VAX1，WDR62，PRKAR2A，PRKAR2B，SMO，STK36，MAPK3 |
