# Supplementary material for: Identification of novel candidate pathogenic genes in pituitary stalk interruption syndrome by whole‐exome sequencing
Source: J Cell Mol Med. 2020 Aug 31;24(20):11703–17. doi: 10.1111/jcmm.15781 (PMC7579688; doi:10.1111/jcmm.15781)
Supplement: Supplementary file 3 — Table S2 [file JCMM-24-11703-s003.docx]

Supplementary Table 2: Mutations validated by Sanger sequence.

| **NO** | **Primer name** | **Patient NO** | **Primer Sequence** | **Results** |
| --- | --- | --- | --- | --- |
| 1 | 1 HESX1-SNV-1F | P49 | TGCAGGAAAGAAAACATCACA | wild type |
|  | 1 HESX1-SNV-1R |  | GCATCCAGATTAATTGCCAAG |  |
| 2 | 2 SOX3-SNV-1F | P21 | CATTTTCGCTGCTCCTGACT | wild type |
|  | 2 SOX3-SNV-1R |  | TGGGCTCGGTAGTGAAGTCT |  |
| 3 | 3 GLI2-SNV-1F | P58 | GTGCTGACCCCTCTGCTCT | validated |
|  | 3 GLI2-SNV-1R |  | AGTGGCTGCCGCGTACTT |  |
| 4 | 4 GLI1-SNV-1F | P11 | GGAAGCTCCTTGACCATCCT | validated |
|  | 4 GLI1-SNV-1R |  | GCATGGGAAGACCACCTATC |  |
| 5 | 5 GLI1-SNV-1F | P5、P39 | GCCCAATCACAAGTCAGGTT | validated |
|  | 5 GLI1-SNV-1R |  | ATGCGATCTGTGATGGATGA |  |
| 6 | 6 PTCH1-SNV-1F | P8 | CACTCCCATGGAAGATGACC | validated |
|  | 6 PTCH1-SNV-1R |  | TGAAACGCAGATTACCATGC |  |
| 7 | 7 PTCH1-SNV-1F | P21 | ATTAGCTGGTGGGAGGAGGT | validated |
|  | 7 PTCH1-SNV-1R |  | TCCTTCTGGCTGCGAGTTAT |  |
| 8 | 8 PTCH1-SNV-1F | P1 | GCAGTCCACTGTCCAGCTC | validated |
|  | 8 PTCH1-SNV-1R |  | CACTCCCATGGAAGATGACC |  |
| 9 | 9 STK36-SNV-1F | P56、P5 | AACAGGATTTGGGGTCCTCT | validated |
|  | 9 STK36-SNV-1R |  | TGTAAGGGAAGTGGCAAAGG |  |
| 10 | 10 STK36-SNV-1F | P20 | CCCTGCTTTCCTTCTCTGTG | wild type |
|  | 10 STK36-SNV-1R |  | GCCCCAGAAGGCATTAGAGT |  |
| 11 | 11 STK36-SNV-1F | P20 | GGGAATGGTACCCTACAGCA | validated |
|  | 11 STK36-SNV-1R |  | TCAGGACAGGCAGAGAGGAT |  |
| 12 | 12 SMO-SNV-1F | P33 | TGCCCAAGTGTGAGAATGAC | validated |
|  | 12 SMO-SNV-1R |  | ATAGACGGATCCAGCCAGTG |  |
| 13 | 13 KIF7-SNV-1F | P57 | TCTAAGGTCACGTGGCTGTG | validated |
|  | 13 KIF7-SNV-1R |  | TGCTGGGATTACAGACGTGA |  |
| 14 | 14 CSNK1A1L-SNV-1F | P1 | GCTGTGCTGCTTTCTGCTTT | validated |
|  | 14 CSNK1A1L-SNV-1R |  | AGGACCAGGCAACACATACC |  |
| 15 | 15 PRKAR2A-SNV-1F | P33、P21 | ACAGCCCTGGACATCAGTTC | validated |
|  | 15 PRKAR2A-SNV-1R |  | GGTCATGTTCTTTGCCTGCT |  |
| 16 | 16 CREBBP-SNV-1F | P4 | CGGTGCTGAGGTAGGAGAAG | validated |
|  | 16 CREBBP-SNV-1R |  | AGAGCCATGCCCATAAGATG |  |
| 17 | 17 GSK3B-SNV-1F | P32 | CCACCACACCTGGCTAAGTT | validated |
|  | 17 GSK3B-SNV-1R |  | GCCCGGCATAAACTGGTAGT |  |
| 18 | 18 ASCL1-indel-1F | P21 | CAGGGCTCCCGCTTCATATT | wild type |
|  | 18 ASCL1-indel-1R |  | CTGTCGCTTGACTTGCTTGG |  |
| 19 | 19 SMAD3-indel-1F | P6 | CTGCGTGAATCCCTACCACT | wild type |
|  | 19 SMAD3-indel-1R |  | AGGGATGCGGTTCTGAGTC |  |
| 20 | 20 MAPK3-indel-1F | P54 | CAGGCCGAAATCACAAATCT | validated |
|  | 20 MAPK3-indel-1R |  | TTAACCAAGTGCCTCCCACT |  |
| 21 | 21 CACNB4-indel-1F | P5 | GCTCCTTGGGTCTCCTCTCT | wild type |
|  | 21 CACNB4-indel-1R |  | TTGCAGGAAAGGAAAAATGG |  |
| 22 | 22 CDON-indel-1F | P5 | TCCTAGAATTCCAATTTGGGGG | wild type |
|  | 22 CDON-indel-1R |  | GGTGAAGGTCCATCCCAGTG |  |
| 23 | 23 FGFR1-Indel-1F | P7、P10 | TGTTAGAGCTTCTCCGCCTC | wild type |
|  | 23 FGFR1-Indel-1R |  | CTTCCTCCTTCCTCAGTGCAT |  |
| 24 | 24 GHRH-Indel-1F | P4、P5 | ACGCTGGAACCCTTTTGCTA | wild type |
|  | 24 GHRH-Indel-1R |  | GGGAAAGTTCCCACCACTCC |  |
| 25 | 25 GNRH2-Indel-1F | P37 | GAAGACAGGCGTGGGGAAAT | validated |
|  | 25 GNRH2-Indel-1R |  | CTTGGGACCTGTAGTCCTCG |  |
| 26 | 26 PROP1-Indel-1F | P34 | AGCCACCCCATTTTCTTGTCT | wild type |
|  | 26 PROP1-Indel-1R |  | CTTCTTACTTGCCCCGAGTGT |  |
| 27 | 27 EGR4-Indel-1F | P48 | GCCTTCAGTGGACTTGACGA | validated |
|  | 27 EGR4-Indel-1R |  | AAGCGCATCTACCGGACG |  |
| 28 | 28 PTCH2-Indel-1F | P37、P50 | CTGGGTAGTGGCAGCATTGA | validated |
|  | 28 PTCH2-Indel-1R |  | GGCAAAAGGCCCTTCACATC |  |
| 29 | 29 CDON-115-1F | P15,P51 | CTGCCTGTGTGGTACCATGT | validated |
|  | 29 CDON-115-1R |  | AGTGTACCTTCAGAAAACTCTCA |  |
| 30 | 30 PRKAR2B-exon9-1F | P34 | AACCGAGACAGCTTGTTCCC | validated |
|  | 30 PRKAR2B-exon9-1R |  | GCCCACTGTTGCAATCCCTA |  |
| 31 | 31 PTCH2-exon11-1F | P55 | TGGGAAGACAAGCATCACGG | validated |
|  | 31 PTCH2-exon11-1R |  | CTTGTCCCCCAGGGCTCATC |  |
| 32 | 32 PTCH2-exon6-1F | P26,P58 | AGGCTTAGAAAAGGAAAAAGGGAT | validated |
|  | 32 PTCH2-exon6-1R |  | ACAGCTCACTGTAGCACCCT |  |
| 33 | 33 GLI2-exon3-1F | P34 | AATACACTATGCTCAGTGCTCA | validated |
|  | 33 GLI2-exon3-1R |  | TTCACAAATGCCACAGGTCC |  |
| 34 | 34 GLI2-exon10-1F | P15,P51 | TTCATGGAGCCCCAAACAGG | validated |
|  | 34 GLI2-exon10-1R |  | CAGGAACTTGCTCTCCTCGG |  |
| 35 | 35 LHX-exon4-1F | P37、P48 | CATATCGTCCCTGCCATCCC | validated |
|  | 35 LHX-exon4-1R |  | TGGGAGGCATTGTGTGAGAC |  |
| 36 | 36 CDON-382-1F | P18,P56 | TGCCAGTGATCCATGCTGTT | validated |
|  | 36 CDON-382-1R |  | AGTCTATTTGTAGTTTCGTAGGGGT |  |
| 37 | 37-NDE1-F | P22 | TCCTAGACACCATGCCACAAG | wild type |
|  | 37-NDE1-R |  | CTGGCACATATAGGCCTGCAA |  |
| 38 | 38-VIPR2-F | P38 | GCCCAGTTTGAATGGGGGTA | wild type |
|  | 38-VIPR2-R |  | GGCAGTGCTGTTCACAGTTG |  |
| 39 | 39-SOX9-F | P11,P25 | CACACCCCAACTCAATCCCA | wild type |
|  | 39-SOX9-R |  | CGGCAGGTACTGGTCAAACT |  |
| 40 | 40-GNAS-F | P34 | CTAGCCCAGCCGAAGAGATG | validated |
|  | 40-GNAS-R |  | TGCGATAACTCGGGAAAGCC |  |
| 41 | 41-HHAT-F | P23 | ATGACCTGGTTTGGGCTCTG | validated |
|  | 41-HHAT-R |  | TGGGAGAGTTGGAATAATACCTTT |  |
| 42 | 42-LRP2-F | P6 | GCATTGGTCCAAACAGCCAAT | wild type |
|  | 42-LRP2-R |  | AACCCGTGACCTACCTAGTGA |  |
| 43 | 43-SPG11-F | P41 | CCCCCACAACAGGGTAATGT | validated |
|  | 43-SPG11-R |  | TCATGACAGGCGATGGCTTT |  |
| 44 | 44-FZD5-F | P4 | CTACCAAACAAAACGCCCCC | wild type |
|  | 44-FZD5-R |  | ACCAGAACCTGAACTCGCTG |  |
| 45 | 45-PTCH2-F | P37 P50 | CTCTCAGGACACTTACCCGC | validated |
|  | 45-PTCH2-R |  | CAACCGAGGCTTCTCCACTT |  |
