# Supplementary material for: Identification of novel candidate pathogenic genes in pituitary stalk interruption syndrome by whole‐exome sequencing
Source: J Cell Mol Med. 2020 Aug 31;24(20):11703–17. doi: 10.1111/jcmm.15781 (PMC7579688; doi:10.1111/jcmm.15781)
Supplement: Supplementary file 4 — Table S3 [file JCMM-24-11703-s004.docx]

**Supplement Table 3.** Frequency and pathogenicity classification of pathogenic variants of PTCH1 and PTCH2

| Gene | Variant | Patient ID | Prediction algorithms | | | | Allele frequency in controls | | |
| --- | --- | --- | --- | --- | --- | --- | --- | --- | --- |
|  |  |  | Polyphen2 | SIFT | Mutation  Taster | CADD  score | 1000 g | ESP6500 | dbSNP |
| Ptch2 | c.1172_1173del:p.S391fs | P37/P50 | . | . | D | . | 0.0005 | . | rs56126236 |
| Ptch2 | c.G788A:p.S263N | P58 | 0.43 | 0.012 | D | 23.3 | . | . | rs77102909 |
| Ptch2 | c.G726C:p.Q242H | P26 | 0.98 | 0.022 | D | 25.6 | . | . | . |
| Ptch2 | c.T311C:p.L104P | P37/P50 | 0.99 | 0.003 | D | 27.7 | 0.0007 | . | rs80168454 |
| Ptch1 | c.C3709T:p.R1237C | P12/P14/P35/P40/P55 | 0.89 | 0.007 | D | 25.4 | 0.0015 | . | rs56102979 |
| Ptch1 | c.C3041T:p.A1014V | P8 | 1 | 0.002 | D | 32 | . | . | . |
| Ptch1 | c.G2480A:p.R827H | P21/P46 | 0.99 | 0.13 | D | 23.7 | 0.0019 | . | rs138154222 |
| Ptch1 | c.C2024T:p.A675V | P1 | 0.94 | 0.06 | D | 27.8 | 0.0009 | . | rs2227971 |
